# From fluid responsiveness to fluid effectiveness: an endothelial perspective on fluid therapy in critical illness

**DOI:** 10.1186/s40560-026-00909-z

**Published:** 2026-07-09

**Authors:** Hideshi Okada, Hiroyuki Tomita, Akio Suzuki, Toshiaki Iba

**Affiliations:** 1 Department of Emergency and Disaster Medicine, Gifu University Graduate School of Medicine, 1-1 Yanagido, Gifu, Japan; 2 Center for One Medicine Innovative Translational Research, Gifu University Institute for Advanced Study, Gifu, Japan; 3 Department of Tumor Pathology, Gifu University Graduate School of Medicine, Gifu, Japan; 4 Department of Pharmacy, Gifu University Hospital, Gifu, Japan; 5 Faculty of Medical Science, Juntendo University, Urayasu, Chiba Japan

**Keywords:** Endothelium, Fluid effectiveness, Fluid responsiveness, Fluid therapy, Glycocalyx

## Abstract

Fluid responsiveness is the cornerstone of circulatory management in critical illness, offering a physiologically intuitive framework for guiding fluid therapy. Nonetheless, clinical experience and accumulating evidence suggest that an increase in cardiac output does not necessarily translate into improved microcirculatory perfusion, particularly during the advanced stages of critical illness. Such dissociation between macrocirculatory stabilization and persistent tissue-level hypoperfusion challenges conventional volume-centered resuscitation paradigms. This review revisits the concept of fluid responsiveness from a microcirculatory perspective and proposes endothelial integrity, rather than circulatory responsiveness alone, as an important determinant of the effectiveness of fluid therapy. By integrating classical physiology with contemporary insights into endothelial biology, this review highlights the endothelial glycocalyx as a functional interface that may influence intravascular fluid retention, vascular permeability, and hemodynamic coherence. Glycocalyx disruption, which is increasingly conceptualized as a component of “endotheliopathy” in critical illness, may be one mechanistic contributor to fluid unresponsiveness, progressive interstitial edema, and diminishing benefits of continued fluid loading. Additionally, this review discusses the implications of this framework for interpreting fluid trials, bedside assessment of tissue perfusion, and endothelial biomarker use to contextualize resuscitation strategies. Building on the established concepts of hemodynamic coherence and the revised Starling-glycocalyx model, this review introduces “fluid effectiveness” as a complementary framework alongside fluid responsiveness, providing a physiologically coherent approach to understanding when fluid therapy can translate into organ-level benefit and why this translation may fail as endothelial and glycocalyx injury progress.

## Background

Fluid responsiveness remains an essential physiological concept for assessing preload reserve and guiding early hemodynamic management. However, accumulating clinical experience and experimental evidence indicate that improvement in cardiac output does not necessarily translate into restoration of tissue perfusion, particularly in the later stages of critical illness. Clinicians frequently encounter patients in whom repeated fluid administration stabilizes macrocirculatory variables, while tissue edema progresses and organ dysfunction persists. Such dissociation challenges the assumption that intravascular volume expansion alone is sufficient to restore effective organ perfusion [1–3]. The concept of fluid responsiveness has been widely adopted, especially during the early phase of resuscitation, based on the premise that administered fluids can be retained within the vascular compartment and effectively transmitted to microcirculation. When this assumption is violated, conventional hemodynamic indices may become dissociated from tissue-level responses. Indeed, improvements in global hemodynamics do not necessarily ensure restoration of microvascular flow, which may remain heterogeneous, intermittently obstructed, or regionally impaired across critical illnesses [4–6].

This macro–microcirculatory dissociation reflects the complex and multifactorial nature of microcirculatory failure, involving not only endothelial dysfunction but also alterations in vascular tone, rheology, cellular metabolism, and regional autoregulatory mechanisms [5, 7]. The dissociation between systemic hemodynamics and microcirculatory perfusion has been previously conceptualized as a loss of hemodynamic coherence, in which correction of macrocirculatory variables fails to restore microcirculatory flow and tissue oxygenation. This concept has provided an important framework for understanding why conventional hemodynamic resuscitation may not always translate into organ-level benefit [8]. Similarly, the revised Starling principle and glycocalyx model have clarified the role of the endothelial surface layer in transvascular fluid exchange and intravascular volume retention [9]. Building on these established frameworks, the present review does not aim to redefine hemodynamic coherence itself, but rather to extend its interpretation in the specific context of fluid therapy. We propose “fluid effectiveness” as a complementary concept that describes whether a hemodynamic response to volume expansion can be translated into meaningful clinical benefit within a given endothelial and microvascular environment.

Within this complex pathophysiology, endothelial injury has emerged as an important contributor to vascular permeability and intravascular fluid retention. The constellation of endothelial barrier disruption, increased permeability, and glycocalyx degradation is increasingly conceptualized as “endotheliopathy”, a syndrome characterized by widespread endothelial dysfunction, loss of barrier integrity, and dysregulated vascular homeostasis. This concept provides a physiological framework linking microvascular dysfunction, interstitial edema, and organ failure [10]. In this context, the endothelial glycocalyx represents a functional interface that may influence whether intravascular volume expansion can be effectively translated into organized microcirculatory flow and tissue oxygen delivery.

**Fig. 1:**
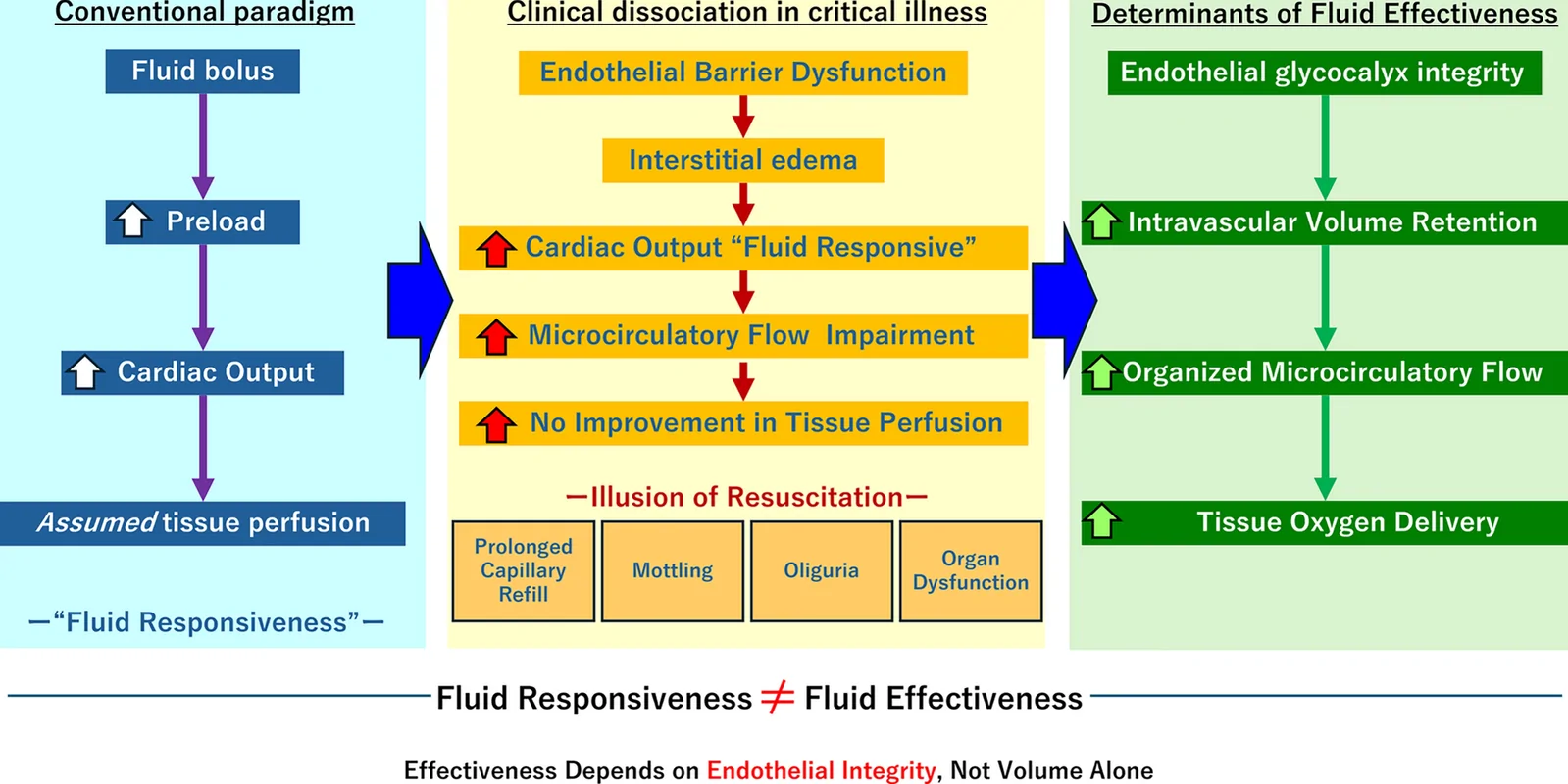
From fluid responsiveness to fluid effectiveness: a microcirculatory perspective. Conceptual framework illustrating the dissociation between fluid responsiveness and fluid effectiveness in critical illness. Fluid administration may increase the cardiac output (left). However, endothelial barrier dysfunction and glycocalyx degradation impair intravascular volume retention and microcirculatory flow, resulting in persistent tissue-level hypoperfusion (center). Thus, effective fluid therapy may depend on endothelial glycocalyx integrity, which contributes to whether intravascular volume expansion translates into meaningful tissue-level perfusion (right)

We therefore propose that the clinical effectiveness of fluid therapy is influenced not only by the amount of administered volume, but also by the functional integrity of the endothelial barrier [11–13]. The conceptual distinction between fluid responsiveness and fluid effectiveness is illustrated in Fig. 1.

## Literature identification

This narrative review was based on a literature search of PubMed covering publications through June 2026. Search terms included “fluid responsiveness”, “fluid therapy”, “microcirculation”, “hemodynamic coherence”, “glycocalyx”, “endothelial dysfunction”, “endotheliopathy”, “vascular permeability”, and “critical illness”. Additional studies were identified through manual review of reference lists from relevant articles. Studies were selected on the basis of their relevance to the physiological relationship between fluid therapy, endothelial function, and tissue perfusion. Because the objective of this review was to develop a conceptual and hypothesis-generating framework, formal systematic-review methodology was not applied.

## Fluid responsiveness in critical illness: what do we think we know?

Fluid responsiveness is defined as the physiological ability of cardiac output to increase in response to intravascular volume expansion and has become a cornerstone concept in the management of critically ill patients. Guided by dynamic hemodynamic indices, this framework has contributed substantially to early resuscitation strategies and short-term circulatory stabilization, particularly during the initial phase of shock [1, 3]. This approach remains embedded in contemporary international recommendations, including the recent European Society of Intensive Care Medicine clinical practice guideline on fluid therapy in adult critically ill patients, which provides evidence-based recommendations regarding fluid resuscitation during the early phase of critical illness [14].

The widespread adoption of fluid responsiveness stems from its physiological plausibility and bedside applicability. By linking preload reserve to measurable changes in cardiac output, clinicians gained a practical tool to rationalize fluid administration in complex and rapidly evolving clinical scenarios [15]. However, this concept implicitly assumes that administered fluids are retained within the intravascular compartment and effectively translate into improved tissue perfusion, assumptions that are rarely examined in routine practice.

Large randomized trials comparing crystalloids and colloids, including the SAFE study, demonstrated little or no difference in mortality despite substantial differences in theoretical intravascular volume expansion [16]. These findings are consistent with the possibility that factors beyond simple intravascular volume expansion influence clinical outcomes in critical illness. However, the mechanisms underlying these observations remain multifactorial and may include differences in fluid distribution, endothelial function, disease severity, and other determinants of microcirculatory perfusion.

Collectively, these observations expose a fundamental uncertainty: even when fluid responsiveness can be demonstrated hemodynamically, why does effective tissue perfusion often fail to follow? Addressing this dissociation requires moving beyond volume-centered metrics toward an integrated evaluation of the microvascular environment and the conditions under which delivered flow can be effectively utilized [5, 17].

## When fluid responsiveness fails: dissociation between hemodynamics and tissue-level perfusion

In daily intensive care practice, clinicians frequently encounter patients who meet conventional criteria for fluid responsiveness yet show no meaningful improvement in tissue perfusion. Despite increases in cardiac output or arterial pressure after fluid administration, markers of organ function and microcirculatory flow may remain unchanged or continue to deteriorate [5, 6, 17]. This mismatch between macrocirculatory stabilization and tissue-level perfusion represents a central limitation of volume-centered resuscitation strategies.

Even when preload responsiveness is preserved, peripheral signs of hypoperfusion—such as prolonged capillary refill time, mottling, oliguria, or impaired organ-specific function—often persist. Metabolic indices may also provide discordant signals; modest reductions in lactate concentration can occur without parallel recovery of microcirculatory flow or organ performance, creating a misleading impression of successful resuscitation [18, 19]. Together, these observations underscore that macrocirculatory improvement does not reliably reflect restoration of tissue perfusion.

As fluid administration continues under these conditions, the clinical problem often shifts from circulatory instability to progressive interstitial edema and secondary organ dysfunction. Pulmonary congestion, impaired gas exchange, rising intra-abdominal pressure, and reduced organ compliance frequently emerge, reflecting cumulative microvascular leakage rather than persistent hypovolemia [20, 21]. Notably, these adverse effects may evolve insidiously and be attributed to disease severity rather than iatrogenic volume burden.

**Fig. 2:**
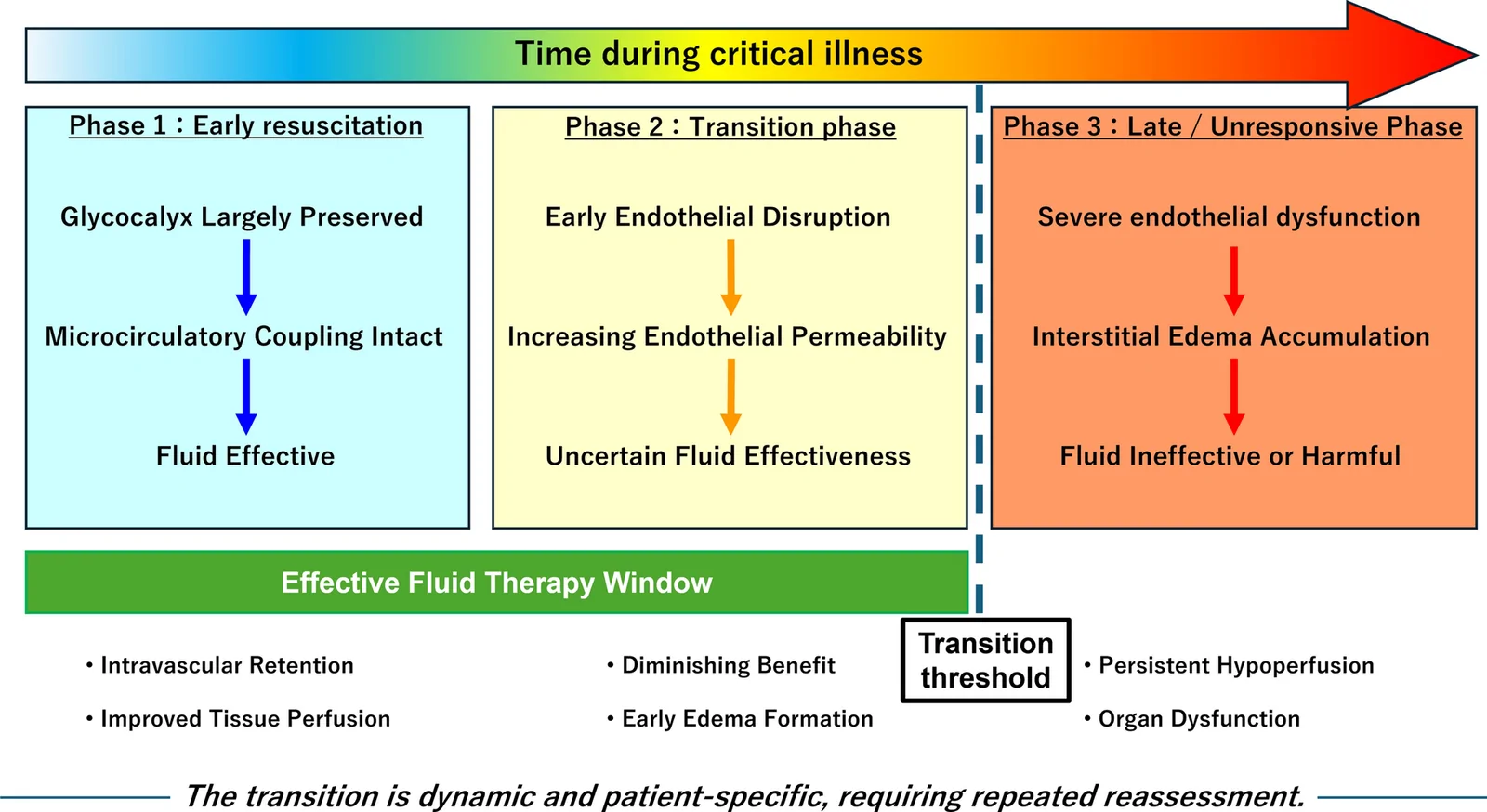
When fluids stop working: time-dependent transition of fluid effectiveness in critical illness. During Phase 1 (early resuscitation), glycocalyx structure is largely preserved, microcirculatory coupling remains intact, and fluid administration is more likely to produce meaningful physiological benefit (“fluid effective”). During Phase 2 (transition phase), progressive endothelial disruption and increasing vascular permeability are associated with uncertain fluid effectiveness. The vertical dashed line represents a conceptual transition threshold beyond which the effectiveness of additional fluid administration progressively declines. During Phase 3 (late/unresponsive phase), severe endothelial dysfunction promotes interstitial edema accumulation, and further fluid administration may become ineffective or potentially harmful. The green bar indicates the conceptual “effective fluid therapy window”, during which fluid administration is most likely to translate into beneficial physiological responses. The figure is intended as a conceptual framework and does not imply a validated clinical threshold

Collectively, these findings describe a state of functional “fluid unresponsiveness”, wherein further volume expansion stabilizes systemic hemodynamics without improving—and potentially worsening—tissue perfusion [3]. Recognition of this transition is critical, as continued fluid loading beyond this point reinforces a self-perpetuating cycle of edema formation and microcirculatory impairment, progressively narrowing the therapeutic window for effective resuscitation. The temporal progression from initial fluid responsiveness to progressive endothelial dysfunction, interstitial edema accumulation, and loss of therapeutic efficacy is summarized in Fig. 2.

## Why fluid administration fails to restore tissue-level perfusion: revisiting the Starling paradigm

The dissociation between macrocirculatory improvement and persistent tissue-level hypoperfusion raises a fundamental question: why does increasing the intravascular volume fail to restore effective perfusion in critical illness? To address this question, the classical assumptions governing transvascular fluid exchange should be revisited, and their validity in systemic inflammation and endothelial injury should be reconsidered.

Conventional fluid resuscitation strategies are implicitly grounded on the classical Starling principle, which conceptualizes capillary fluid exchange as a balance between hydrostatic and oncotic pressures. Within this framework, intravascular volume expansion is expected to augment capillary hydrostatic pressure and facilitate plasma refilling, thereby ultimately improving tissue-level perfusion. Nonetheless, experimental and clinical evidence suggest that these assumptions no longer hold in critical illness [22, 23].

### Limitations of the classical Starling principle in critical illness

In critical illness, however, the structural and functional properties of the endothelial barrier are profoundly altered by inflammation, ischemia–reperfusion injury, and mechanical stress. Increased permeability permits plasma proteins to escape into the interstitial space, attenuating the effective oncotic gradient and reducing the capacity of administered fluids to be retained intravascularly. Resultantly, additional volume expansion increasingly favors interstitial accumulation rather than sustained plasma volume support [9, 22].

Importantly, experimental and clinical data indicate that capillary reabsorption is minimal under most physiological conditions and becomes even more limited during systemic inflammation. Elevations in capillary hydrostatic pressure therefore predominantly accelerate net filtration, promoting tissue edema rather than plasma refilling [24–26]. This revised understanding challenges the assumption that intravascular volume expansion reliably translates into improved tissue perfusion in critical illness.

These limitations suggest that the failure of fluid therapy cannot be explained by circulatory mechanics alone. Instead, disruption of the endothelial interface itself becomes an important determinant of fluid distribution and therapeutic effectiveness, providing a physiological basis for re-examining the role of the endothelial glycocalyx in microvascular regulation [24, 27].

### Endothelial glycocalyx as a functional barrier

The inability of fluid administration to restore effective tissue perfusion in critical illness cannot be attributed solely to inadequate intravascular volume expansion. Increasing evidence indicates that this dissociation reflects a fundamental alteration of the endothelial interface governing transvascular fluid exchange. Within this framework, the endothelial glycocalyx functions as an important regulator linking macrocirculatory flow to microcirculatory perfusion [9, 11, 22].

Rather than serving merely as a structural coating, the glycocalyx modulates endothelial permeability and preserves an effective intravascular–interstitial protein gradient at the luminal surface. Disruption of this layer alters protein distribution, weakens barrier function, and promotes unrestrained filtration into the interstitium. As a result, increases in capillary hydrostatic pressure preferentially drive fluid extravasation rather than supporting intravascular volume retention or microvascular perfusion [12, 28].

In critical illness, the glycocalyx is particularly susceptible to inflammatory signaling, ischemia–reperfusion injury, mechanical stress, and excessive fluid exposure. Experimental and clinical studies consistently demonstrate rapid shedding and functional degradation of glycocalyx components under these conditions, accompanied by increased vascular permeability and tissue edema [13, 29, 30].

Glycocalyx disruption may provide a mechanistic framework that helps explain several clinical observations discussed earlier. Preload responsiveness may persist despite impaired tissue perfusion because cardiac output augmentation occurs within a vascular environment incapable of effectively retaining or distributing intravascular volume. Likewise, the diminishing efficacy of repeated fluid loading and the development of progressive edema reflect loss of the microvascular barrier required for coherent flow transmission and oxygen delivery [4, 5, 31].

Thus, the endothelial glycocalyx represents not merely an anatomical structure but a functional interface that may influence whether intravascular volume expansion can translate into meaningful tissue perfusion. Once this barrier is compromised, fluid therapy may stabilize macrocirculatory parameters while simultaneously exacerbating microcirculatory dysfunction, underscoring the need to interpret fluid responsiveness within the context of endothelial integrity [10].

### Clinical implications: beyond volume expansion

Recognizing glycocalyx dysfunction has important implications for fluid therapy in critical illness. Disruption of the endothelial barrier required for translating intravascular volume expansion into effective tissue-level perfusion may fundamentally limit the clinical effects of additional fluid administration. Under such conditions, the conventional focus on optimizing preload and cardiac output alone is insufficient to ensure the restoration of microcirculatory flow.

Large randomized controlled trials comparing crystalloids and colloids, including albumin-based solutions, have consistently demonstrated limited differences in patient-centered outcomes [16]. From the perspective of the proposed framework, these findings are consistent with the possibility that endothelial barrier dysfunction may limit the capacity of both crystalloids and colloids to produce sustained tissue-level benefit. However, this interpretation is not specific to glycocalyx dysfunction, and alternative explanations—including differences in fluid distribution, disease severity, chloride load, and broader mechanisms of microcirculatory dysfunction—may also contribute to the observed outcomes.

Beyond SAFE, findings from other major fluid-therapy trials provide an opportunity to critically examine both the explanatory value and the limitations of the proposed framework, while further highlighting the complexity of the relationship between fluid administration and clinical outcomes. ALBIOS and CRISTAL evaluated albumin-containing resuscitation strategies, whereas SMART, BaSICS, and PLUS compared balanced crystalloids with saline-based approaches [32–36]. Although these studies were not designed to directly assess endothelial integrity or glycocalyx function, their heterogeneous findings suggest that the effectiveness of fluid therapy depends on factors extending beyond simple volume expansion. Differences in fluid composition, vascular permeability, microcirculatory function, and patient-specific biological context may all influence the translation of hemodynamic responses into clinical benefit.

This framework does not negate the value of fluid therapy. During the early phase of critical illness, when the glycocalyx and microvascular environment may still be partially preserved, volume expansion can improve macrocirculatory stabilization and support initial resuscitation. However, the therapeutic window for effective fluid administration narrows as endothelial dysfunction progresses. Beyond this window, continued volume loading may preferentially exacerbate interstitial edema without conferring additional benefits at the tissue level. The clinical consequences of interstitial fluid accumulation are influenced not only by vascular leakage but also by the capacity of the lymphatic system to remove excess interstitial fluid. Although a detailed discussion of lymphatic physiology is beyond the primary scope of this review, impaired lymphatic clearance may further contribute to edema persistence and reduced fluid effectiveness [37–39].

Accordingly, the clinical challenge shifts from ascertaining the amount of fluids to administer toward determining when fluid therapy is likely to lose efficacy. Assessment of tissue-level perfusion, signs of fluid unresponsiveness, and ongoing organ dysfunction is central to this decision-making process. In this context, restraint on fluid administration should not be interpreted as therapeutic nihilism, but as a recognition of altered vascular physiology.

Ultimately, a glycocalyx-centered perspective reframes fluid resuscitation as a context-dependent intervention, the effectiveness of which is contingent on microvascular barrier integrity. Rather than abandoning fluid therapy, this approach emphasizes the necessity of integrating fluid administration with strategies that preserve or restore the microcirculatory environment where fluids are expected to act.

## Mechanistic basis of fluid effectiveness

The clinical effectiveness of fluid therapy is likely influenced by multiple physiological factors beyond the amount and type of administered fluids, including the vascular environment into which these fluids are delivered. From this perspective, endothelial glycocalyx integrity may contribute to the translation of intravascular volume expansion into tissue-level perfusion, although this relationship remains incompletely understood and is likely influenced by additional determinants of microcirculatory function [11, 12].

As discussed above, preservation of glycocalyx integrity may facilitate intravascular volume retention and coherent microcirculatory flow, whereas glycocalyx disruption may favor interstitial fluid accumulation and reduced fluid effectiveness [22].

This framework aids in reconciling the apparent inconsistency between hemodynamic responsiveness and clinical outcomes in critical illness. Fluid responsiveness at the cardiac level may persist despite profound endothelial dysfunction, creating the illusion of effective resuscitation even when tissue-level perfusion remains impaired. Thus, fluid responsiveness and endothelial integrity should be regarded as complementary physiological determinants of fluid therapy. While fluid responsiveness reflects preload reserve and the likelihood of cardiac output augmentation, endothelial integrity may influence whether this hemodynamic response can be translated into meaningful tissue-level benefit.

Recent clinical discussions have further highlighted the interaction between resuscitation fluids and endothelial function, including the potential role of albumin in modulating endothelial permeability and glycocalyx integrity, reinforcing the concept that fluid composition alone cannot overcome endothelial dysfunction [40].

Endothelial integrity provides a physiological context for interpreting fluid effectiveness. This perspective shifts the clinical focus from the selection of optimal fluids toward a broader consideration of the physiological conditions under which fluid therapy is most likely to be beneficial. In this sense, fluid administration may be regarded as a context-dependent intervention, the effectiveness of which is influenced not only by administered volume but also by microvascular barrier integrity and other determinants of tissue perfusion. Recognizing the potential role of the glycocalyx in fluid effectiveness may help bridge the gap between microcirculatory physiology and bedside decision-making.

Several additional mechanisms may contribute to dissociation between macrocirculatory augmentation and tissue-level perfusion in critical illness. These mechanisms include alterations in blood rheology, vasomotor dysregulation, distributive microvascular shunting, mitochondrial dysfunction, and organ-specific heterogeneity in microvascular responses. This review does not propose endothelial glycocalyx dysfunction as the sole explanation for impaired fluid effectiveness; rather, it highlights endothelial integrity as an important physiological context that influences the translation of macrocirculatory flow into effective tissue perfusion.

## Endothelial integrity as a determinant of fluid effectiveness

In the present review, the term “endothelial integrity” refers to the functional integrity of the endothelial barrier, including preservation of glycocalyx structure, regulation of vascular permeability, and maintenance of microvascular homeostasis. Because no direct bedside measure of endothelial integrity currently exists, its assessment remains dependent on indirect physiological and biomarker-based surrogates.

Importantly, currently available biomarkers of glycocalyx injury, including syndecan-1 and heparan sulfate, should be interpreted cautiously. Although elevated concentrations are associated with endothelial injury, vascular dysfunction, and adverse outcomes in critical illness, these markers may also reflect overall disease severity and systemic inflammation. Therefore, current biomarker data are primarily associative and do not establish a direct causal relationship between glycocalyx shedding and impaired fluid effectiveness.

Fluid responsiveness has traditionally been defined as the ability of the heart to augment cardiac output in response to volume expansion [3, 15]. While this concept provides a pragmatic framework for bedside decision-making, it implicitly assumes that increases in macrocirculatory flow translate into improved tissue perfusion [5]. As discussed in preceding sections, this assumption frequently fails in critical illness [4, 6, 19].

From a microcirculatory perspective, the clinical relevance of fluid responsiveness depends not only on cardiac preload reserve but also on the capacity of the endothelial interface to distribute and retain intravascular volume in a functionally meaningful manner [11, 12, 41]. A positive hemodynamic response may therefore indicate preserved cardiac responsiveness while offering limited information about the ability of the microvasculature to utilize the delivered flow [4, 5].

Endothelial integrity provides a physiologically coherent framework for understanding why fluid effectiveness may diminish as critical illness progresses, even when fluid responsiveness remains preserved. An endothelium capable of maintaining barrier integrity and microvascular coherence may facilitate the coupling of macrocirculatory augmentation to tissue oxygen delivery [9, 13, 42]. Conversely, when endothelial function is impaired, additional volume may increase cardiac output without improving—and potentially worsening—tissue-level perfusion [21].

Importantly, this reframing does not replace established dynamic indices of preload responsiveness but contextualizes their interpretation by recognizing that hemodynamic responsiveness alone is insufficient to infer effective resuscitation [1, 3, 15]. Dynamic indices remain useful to determine whether the heart can respond to volume expansion; however, they should be integrated with concurrent assessment of tissue perfusion and endothelial vulnerability [6, 11].

Clinically, this perspective shifts the central question from whether cardiac output will rise to whether the vascular environment can translate that increase into tissue benefit. Recognizing this distinction may help avoid continued volume loading in patients who remain technically fluid responsive but functionally fluid unresponsive at the microcirculatory level [10].

Although no validated glycocalyx-guided algorithm or decision threshold currently exists, the concept of fluid effectiveness may provide a provisional framework for integrating information already available in routine clinical practice. Such an approach may combine assessment of fluid responsiveness, tissue perfusion, fluid accumulation, and markers of endothelial vulnerability to contextualize decisions regarding ongoing fluid administration. A proposed hypothesis-generating bedside framework is shown in Table 1.

**Table 1:** Integrating fluid responsiveness, tissue perfusion, and endothelial vulnerability in bedside fluid assessment

STEP	Assessment	Clinical implication
1	Fluid responsiveness	Can cardiac output increase?
2	Tissue perfusion (CRT, lactate trajectory, urine output)	Is tissue perfusion improving?
3	Endothelial vulnerability (fluid balance, edema, biomarkers)	Is further fluid administration likely to remain beneficial?
4	Reassessment	Continue, limit, or discontinue fluid administration?

The questions shown in this table are intended to support clinical reasoning and should not be interpreted as validated decision thresholds or treatment algorithms

## Biomarkers and bedside implications

Translating a glycocalyx-centered framework into clinical practice requires indicators that reflect the functional state of the endothelial and microvascular environment. Circulating biomarkers of endothelial injury and glycocalyx shedding have therefore attracted interest, not as therapeutic targets, but as contextual signals of vascular vulnerability [5, 11, 12].

Among these, circulating glycocalyx components, such as syndecan-1 and heparan sulfate fragments, are consistently elevated in systemic inflammation, ischemia–reperfusion injury, and critical illness [29, 30, 42, 43]. Higher concentrations correlate with disease severity and adverse outcomes, supporting their role as surrogate indicators of endothelial disruption rather than direct determinants of therapeutic response [20, 30, 42, 44].

From a fluid management perspective, elevated glycocalyx-derived biomarkers indicate a vascular environment in which intravascular volume is unlikely to be effectively retained and additional fluid is prone to extravasation [9, 21]. In such settings, a positive hemodynamic response may coexist with limited microcirculatory utilization of delivered flow, consistent with the concept of functional fluid unresponsiveness discussed earlier [4].

Biomarker assessment alone, however, is insufficient for bedside decision-making. Circulating levels reflect global shedding and lack organ-level resolution. Temporal variability, interindividual heterogeneity, and the absence of validated thresholds further restrict their use as standalone decision tools. Accordingly, biomarker data should be interpreted alongside clinical assessment of tissue perfusion and evolving organ function rather than in isolation [18].

Ultimately, the value of endothelial biomarkers lies in refining physiological interpretation rather than dictating therapy. Rather than serving as independent triggers, glycocalyx-related signals modify expectations of how the circulation will respond to fluid administration. Identical fluid challenges may, therefore, yield fundamentally different tissue-level effects depending on endothelial vulnerability. When integrated with hemodynamic indices and bedside perfusion assessment, these markers may help identify patients in whom continued volume expansion is unlikely to confer benefit and may instead increase the risk of cumulative tissue edema [1].

Although no validated glycocalyx-guided algorithm currently exists, integrating endothelial and tissue perfusion-related variables may offer a broader physiological framework for interpreting fluid effectiveness in critically ill patients **(**Table 2**)**.

**Table 2:** Conventional hemodynamic assessment and expanded physiological interpretation of fluid effectiveness

Conventional approach	Expanded physiological interpretation
Fluid responsiveness	Preload reserve
Capillary refill	Tissue perfusion context
Lactate	Metabolic stress marker
Endothelial biomarkers	Potential indicator of vascular vulnerability
Edema progression	Possible sign of impaired fluid effectiveness

## Limitations and future perspectives

Despite the conceptual coherence of a glycocalyx-centered framework, several important limitations must be acknowledged. Endothelial dysfunction represents only one component of the complex pathophysiology governing microcirculatory failure in critical illness. Alterations in vascular tone, rheological blood properties, mitochondrial dysfunction, and organ-specific microvascular regulation contribute to the heterogeneity of tissue-level perfusion and cannot be fully captured by glycocalyx integrity alone [5, 7].

Moreover, the assessment of endothelial and glycocalyx status in clinical practice remains indirect. Circulating biomarkers reflect systemic shedding but do not provide spatial or temporal resolution at the level of individual organs. Similarly, bedside indicators of tissue-level perfusion are influenced by multiple factors and may not exclusively represent endothelial integrity. These limitations underscore the challenge of translating microvascular concepts into precise, real-time clinical decision tools [31].

Another important consideration is the dynamic nature of endothelial injury and recovery. Glycocalyx disruption is not a static state, and its extent and reversibility likely vary across disease stages, organs, and therapeutic interventions. Accordingly, the effectiveness of fluid therapy may evolve over time within the same patient, emphasizing the need for repeated reassessment rather than fixed resuscitation strategies.

Thus, future studies should focus on integrative approaches combining endothelial biomarkers, microcirculatory monitoring, and clinical perfusion assessment. Advances in bedside imaging of the microcirculation, improved characterization of lymphatic function, and exploration of strategies aimed at preserving or restoring endothelial integrity represent promising directions. Such efforts may enable more individualized resuscitation strategies that move beyond uniform volume-based protocols [37, 45].

Ultimately, reframing fluid responsiveness and fluid effectiveness through the lens of endothelial integrity does not offer a simple solution, but rather a more physiologically consistent framework. By recognizing the conditions under which fluid therapy loses efficacy, clinicians may better balance the benefits and risks of volume expansion. In this sense, the future of fluid resuscitation may lie not in administering more precise volumes, but in understanding when fluids are still able to exert beneficial effects within a compromised microvascular environment.

In addition, endothelial dysfunction affects vascular leakage and downstream clearance of extravasated fluid. Emerging evidence suggests that lymphatic dysfunction may further exacerbate interstitial edema and prolong microcirculatory failure. Although beyond the scope of this review, integrating lymphatic function into microcirculatory assessment may facilitate an understanding of fluid intolerance in critical illness [38, 39].

Finally, endothelial dysfunction affects not only vascular permeability but also downstream interstitial fluid clearance. Future studies integrating endothelial and lymphatic physiology may further refine the concept of fluid effectiveness. Accordingly, the concepts discussed in this review should currently be considered hypothesis-generating rather than clinically validated decision-making frameworks.

## Conclusions

Fluid responsiveness has long served as a practical framework for assessing preload reserve during fluid therapy in critically ill patients. However, accumulating clinical and experimental evidence indicates that an increase in cardiac output does not necessarily translate into restoration of tissue-level perfusion, particularly as critical illness progresses. This dissociation highlights a fundamental limitation of volume-centered resuscitation strategies.

In this review, we propose that the effectiveness of fluid therapy is influenced not only by circulatory responsiveness but also by endothelial integrity. In particular, endothelial glycocalyx integrity may contribute to the translation of intravascular volume expansion into microcirculatory flow and tissue oxygen delivery, although this relationship remains incompletely understood and is likely influenced by multiple physiological factors. Once glycocalyx disruption and endotheliopathy are established, additional fluid administration may stabilize macrocirculatory parameters while simultaneously promoting interstitial edema and microcirculatory dysfunction.

This conceptual distinction between fluid responsiveness and fluid effectiveness provides a physiologically coherent explanation for why fluid therapy is beneficial during the early phase of critical illness but progressively loses efficacy as endothelial injury advances. Importantly, a positive hemodynamic response should not be equated with effective resuscitation when the microvascular environment is no longer capable of utilizing delivered flow.

The key clinical message of this review is that fluid therapy should be interpreted as a context-dependent intervention. Bedside decision-making must integrate hemodynamic indices with assessment of tissue-level perfusion and the endothelial state, rather than relying on circulatory responsiveness alone. Biomarkers of glycocalyx degradation, together with simple clinical signs of perfusion, may help identify patients in whom further fluid loading is unlikely to confer benefit.

Future advances in fluid management may depend not only on improving the precision of hemodynamic monitoring, but also on developing strategies that preserve or restore endothelial integrity. Whether protection of the endothelial glycocalyx can prolong the period during which fluid therapy remains effective represents an important hypothesis requiring prospective investigation [46].

Ultimately, integrating endothelial integrity into the interpretation of fluid responsiveness shifts the focus of resuscitation from “how much fluid to give” to “when fluids can meaningfully work”. This perspective emphasizes that the future of fluid management in critical illness lies not in increasingly precise volume administration, but in understanding the vascular context in which fluids are expected to act.

## Data Availability

No datasets were generated or analyzed during the current study.
